# Automated Feature Extraction in Brain Tumor by Magnetic Resonance Imaging Using Gaussian Mixture Models

**DOI:** 10.1155/2015/868031

**Published:** 2015-06-02

**Authors:** Ahmad Chaddad

**Affiliations:** Department of Diagnostic Radiology, University of Texas MD Anderson Cancer Center, 1400 Pressler Street, Houston, TX 77030, USA

## Abstract

This paper presents a novel method for Glioblastoma (GBM) feature extraction based on Gaussian mixture model (GMM) features using MRI. We addressed the task of the new features to identify GBM using T1 and T2 weighted images (T1-WI, T2-WI) and Fluid-Attenuated Inversion Recovery (FLAIR) MR images. A pathologic area was detected using multithresholding segmentation with morphological operations of MR images. Multiclassifier techniques were considered to evaluate the performance of the feature based scheme in terms of its capability to discriminate GBM and normal tissue. GMM features demonstrated the best performance by the comparative study using principal component analysis (PCA) and wavelet based features. For the T1-WI, the accuracy performance was 97.05% (AUC = 92.73%) with 0.00% missed detection and 2.95% false alarm. In the T2-WI, the same accuracy (97.05%, AUC = 91.70%) value was achieved with 2.95% missed detection and 0.00% false alarm. In FLAIR mode the accuracy decreased to 94.11% (AUC = 95.85%) with 0.00% missed detection and 5.89% false alarm. These experimental results are promising to enhance the characteristics of heterogeneity and hence early treatment of GBM.

## 1. Introduction

Providing quantitative and accurate information for medical diagnosis, Magnetic Resonance Imaging (MRI) plays an essential role in medical imaging [1]. MRI has several advantages over other medical imaging techniques regarding its multiple applications, namely, for cardiovascular, musculoskeletal, and, in particular, for imaging of the brain and neurological systems [2, 3]. However, a bottleneck of MR image processing arises from variations in intensity due to B1 and B0 field inhomogeneity [4, 5]. This is manifested by the nonuniform appearance even of a single tissue which may mislead image analysis algorithms, which enhance abnormality area detection by a segmentation model [2, 6].

In the last decade, MR imaging established itself as key imaging modality in diagnosis and follow-up of brain tumors including Glioblastoma (GBM) [7]. GBM is the most common primary malignant brain tumor in adults [8]. It is characterized by abnormal and uncontrolled cell proliferation, necrosis, and vascular proliferation [9]. Despite the ongoing research and clinical trials, GBM remains one of the most aggressive malignant tumors with less than 5% of patients surviving five years after diagnosis [10]. This is attributed to the highly infiltrative nature and the heterogeneity that Glioblastoma exhibits on molecular and genomic levels which lead to differences in individual treatment response and prognosis [11].

Accordingly, research has focused on exploring associations between certain imaging features and the underlying genomic profiles of GBM in a new branch in clinical radiology known as “imaging genomics” [12, 13]. Using a GMM, Simon et al. recently showed that delineation and quantification of apparent diffusion coefficient in gliomas can be performed reliably and fast and demonstrated how thereby user-dependent variability can automatically be removed [14]. Consequently, recent work has focused on developing robust methods for reading and imaging features extraction from such MR images.

Automatic reading algorithms can foster faster and more precise readings of MR images as well as segmenting the abnormal imaging areas to classify them as GBM or not. Robust reading of MR images includes several consecutive steps. The system must first segment the image by detecting and extracting the abnormal area from their surrounding medium using multithresholding segmentation and morphological image processing. This step requires careful selection of the appropriate segmentation methodology for processing of high resolution grayscale MR images. While several segmentation methods based on MR images have been proposed using filtering to remove noise, these techniques are not generally applicable to automated detection of GBM as the tumor can be unintentionally eliminated during the process of noise reduction. Segmentation methods based on thresholding or multithresholding are thus preferred.

That way, it is likely that GBM and the normal brain tissue “survive” the thresholding. This method divides an image into several regions using multithresholding [15–18]. The second step following the detection of area of imaging abnormality, representing GBM, involves extraction of some characteristic parameters and texture features that are specific for GBM [19–26]. Plurals based on the texture features were proposed where the visual analysis of texture is a difficult task, particularly with GBM. The texture analysis based on gray level cooccurrence matrix (GLCM) determines neighborhoods around pixels (texture elements) where the GLCM is counted using the specific offset and phases [27]. Also, shape and texture feature were used to classify the brain tumor type and grade using SVM model; however the classifier accuracy was limited by 85% [28].

Moreover, the feature quality is essential to improve the classifier accuracy and accordingly the applications. For example, wavelet based classification has proven to be a powerful technique [29–31]. However, due to its comparatively low classification accuracy this approach was not promising to follow in our MRI data. We therefore aimed to investigate GBM tumor features that may have the potential to measure specific GBM characteristics. To achieve this, we focused on features derived from Gaussian mixture model (GMM) analysis on both weighted T1 and T2 and FLAIR sequences.

**Figure 1:**
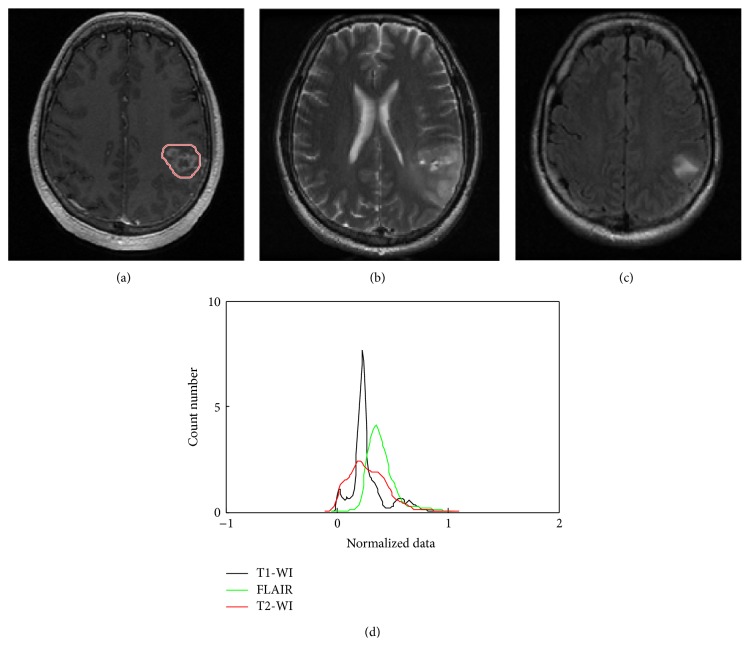
Analysis of GBM schema: (a) brain tumor image on axial T1-WI, (b) axial T2-WI, (c) axial FLAIR sequence, and (d) GBM data fitting in three MR sequences.

Figure 1 shows 2D axial image of brain within GBM region indicated by the red line in Figure 1(a). Clearly, GBM area has higher intensity on the grayscale level brain MR image, but some pixels of normal brain share the same intensity values as the GBM pixels. These pixels closely resemble the GBM pixels in terms of their intensity, rendering GBM detection a difficult task. Also, histogram of GBM area is not similar in the three MRI sequences.

## 2. Materials and Methods

**Figure 2:**
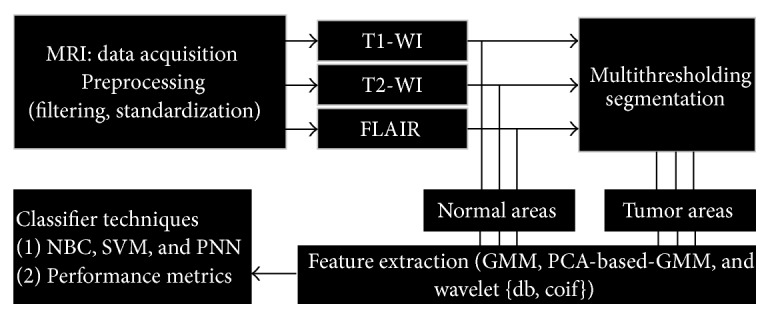
Schematic diagram of the proposed method for automatic feature extraction.

The schematic of the proposed method is shown in Figure 2: (1) preprocessing to normalize grayscales and filtering to remove the noise from images in the three MRI sequences, T1-WI, T2-WI, and FLAIR; (2) tumor (GBM) areas detection by multithresholding segmentation and normal areas determined from the normal brain material; (3) feature extraction from the GMM curve fitting of the grayscale histogram on T1-WI, T2-WI, and FLAIR images; (4) applying three classifier techniques to discriminate between the tumor areas and normal areas based on GMM features; and (5) validating the effect of GMM features by comparative study with PCA and wavelet features. The details of the schematic are given below.

### 2.1. Data Acquisition

A data set of 17 patients was collected by November 2013 from the publicly available Cancer Imaging Archive (http://www.cancerimagingarchive.net/) database for our preliminary study. We excluded patients with incomplete imaging data set. All of the images had 512 × 512 pixels acquisition matrices and were converted into grayscale before further processing. MRI raw data were filtered to remove noise and standardized by the linear normalization, followed by multithreshold-based segmentation. This technique was applied to determine the tumor position and was successfully applied for the GBM data collection process. Note that preprocessing of skull stripping is required; it is necessary to obtain only the brain material without the skull bone; however, multithresholding segmentation with morphological operation filter may be detecting GBM area in two-dimension axial image. In this context, automated operation can be a difficult task if the GBM area is smaller than skull thickness.

### 2.2. Multithresholding Segmentation

Single threshold segmentation for GBM region pixels may resemble normal brain pixels. Segmentation based on multithresholding resolved this problem. Accordingly, we carried out an initial estimation of GBM localization by using multithresholding (multilevel image thresholds) segmentation method proposed by Otsu [32]. This approach enabled the definition of thresholds that maximize the interclass variances, thus also minimizing the intraclass variances. It can offer multilevel image thresholds in order to segment the desired object (brain tumor). In our case, we adjusted the multithresholding of an image for skull stripping and tumor detection. In order to robustly detect GBM, we had to resolve the problem arising from resembling pixels spots. This could be easily resolved by median filter depending on the window size.

### 2.3. GMM Feature Extracted

Many GMM had previously been considered in the literature for face identification [27], which was found to offer the best trade-off in terms of complexity, robustness, and discrimination. It has also been used for voice identification based on the feature and score normalization techniques [33, 34]. Also, GMM based features show promise accuracy classifier to distinguish between target and ghost/clutter regions [35].

GMM is a parametric probability density function represented as a weighted sum of *K* Gaussian component densities according to

(1) $$\begin{array}{c} p\left(\;x\setminus \lambda \;\right)=\overset{K}{\underset{i=1}{\sum }}w_{i}g\left(\;x\setminus \mu_{i},\Sigma_{i}\;\right), \end{array}$$

where *x* represented the *N* dimensional continuous valued data vector, *w*_*i*_ the mixture weight, and *g* the component Gaussian densities.

Each component density was controlled by the *N*-variate Gaussian function according to

(2) gx∖μi,Σi=12πN/2Σi1/2exp⁡−12x−μiTΣi−1x−μi,

where *μ*_*i*_ was the average of a vector, *T* is the transpose, and Σ_*i*_ is the covariance matrix.

The complete Gaussian mixture model was parameterized by the mean vectors, covariance matrices, and mixture weights from all component densities. These parameters can be expressed according to

(3) $$\begin{array}{c} \lambda =\left\{\;w_{i},\mu_{i},\Sigma_{i}\;\right\}. \end{array}$$

The variance (*v*_*i*_) of components was represented by the diagonal of the covariance matrix Σ_*i*_. We extracted then a feature vector *R* from three components of GMM according to

(4) $$\begin{array}{c} R=\left\{\;w_{1}\cdots w_{3},\mu_{1}\cdots \mu_{3},v_{1}\cdots v_{3}\;\right\}, \end{array}$$

where *w*, *µ*, and *v* are the weight, average, and variance of GMM components (indexes 1, 2, and 3 are the first, second, and third component of GMM).

Each segmented GBM area could be represented by the feature vector *R*_GBM_ that is of size 1 by 9 elements. A similar feature vector size (*R*_*N*_) for normal area was computed.

For *n* GBM areas, we had *Rt*_GBM_ matrix that was of size *n* by 9 elements, meaning *n* samples. Similar matrix for the area of normal brain was *Rt*_*N*_. When computing the matrixes *Rt*_GBM_ and *Rt*_*N*_, the classification operation became ready.

### 2.4. Principal Component Analysis Applied on GMM Features

In the following, we present a principal component analysis technique to reduce the data and to get the appropriate feature from each vector feature.

Each feature vector of GBM and of normal brain was extracted from several Gaussian distributions which were represented by the average, standard deviation, and weight. Concatenating the parameters of GMM, this technique could show the correlation between the features extracted. Further, it could have been a good factor classifier to distinguish between GBM and normal brain tissue. Two matrixes (*Rt*_GBM_) and (*Rt*_*N*_) of *n* GBM and normal samples were *n* by 9, where each feature row concerns 9 elements. GBM and normal area samples of *n* = 17 patients were arranged into data matrixes *Rt*_GBM_ and *Rt*_*N*_ according to

(5) RtGBM=R1G⋯RnGRtN=R1N⋯RnN,

where [*R*_1*G*_ ⋯ *R*_*nG*_] and [*R*_1*N*_ ⋯ *R*_*nN*_] were the GMM features of GBM and normal area, respectively.

Training data were received by *Rt*_GBM_ and *Rt*_*N*_. PCA was employed, where the covariance of *Rt*_GBM_ and *Rt*_*N*_ was computed. The covariance matrix could be found according to

(6) CGBM=cov⁡RtGBM,CN=cov⁡RtN,

where cov was the covariance. *C*_GBM_ and *C*_*N*_ are the same size 9 by 9.

According to the following equation, the eigenvalues and eigenvectors could be computed according to

(7) $$\begin{array}{c} CV=\Lambda V, \end{array}$$

where *V* was the matrix of principal component and each column in *V* was an eigenvector. Λ was the diagonal matrix where the diagonal elements were the values of the eigenvalues.

**Figure 3:**
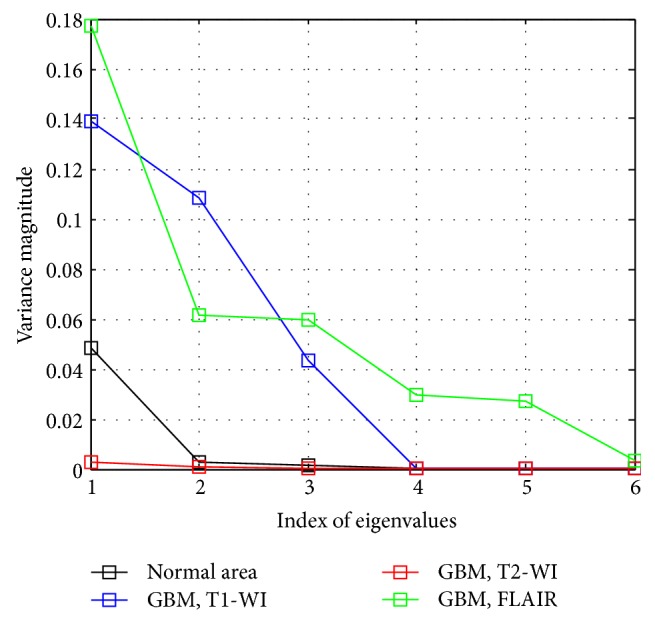
Principal components based on higher variance of GBM and normal areas: (black curve) variance magnitude of 17 normal areas from T1-WI, T2-WI, and FLAIR, (blue curve) variance magnitude of 17 GBMs chosen from T1-WI, (red curve) variance magnitude of 17 GBMs chosen from T2-WI, and (green curve) variance magnitude of 17 chosen GBMs chosen from FLAIR mode of MRI.

We organized the eigenvectors by their corresponding eigenvalues and we retained three eigenvectors as the PCs of the data from *C*_GBM_ and *C*_*N*_, respectively, where the higher variance represented the three largest eigenvalues.  Figure 3 shows the variance of eigenvalues of three MRI modes. Clearly, the maximum variances common between T1-WI, T2-WI, and FLAIR were located in the first three indexes of eigenvalues. The matrix dimension of *Rt*_GBM_ and *Rt*_*N*_ was reduced by the projection of each row according to

(8) PiGBM=PCGBMTRtGBM,PiN=PCNTRtN,

where { }^*T*^ was the transpose indicator, PC_GBM_^*T*^ and PC_*N*_^*T*^ were the transpose of principal components of GBM and normal area, respectively, and *i* was the index of row in GBM matrix *Rt*_GBM_ and normal area matrix *Rt*_*N*_, respectively.

Using 3 PCs, a new matrix *P* ^GBM^ of GBM that was of size 17 by 3 and matrix *P* ^*N*^ of normal area had a similar size. We considered then three classifier models to evaluate GBM and normal areas discrimination based on GMM features and their three principal components, respectively.

### 2.5. Classifier Setting

In general, the goal of a learning/classification algorithm is to build a set of training examples with class labels. In this context, we implemented three classifier techniques, namely, naïve Bayes (NB) [36], support vector machine (SVM) [37], and probabilistic neural network (PNN) [38]. The implementation of NB is performed using a kernel estimation method which approximated the complex distributions of the data. Then, SVM was implemented using the Gaussian radial basis function, and radial basis network based PNN was employed which is a fast classifier technique. The reason for using these specific classifier methods is to achieve the trade-off performance which is reported.

Due to the limited data available (17 patients), validation data sets were performed based on leave-one-out cross-validation [39]. Performance metrics are expressed by the following equations:

(9) False  Alarm=number  of  normal  samples  uncorrectly  classifiedtotal  number  of  sample  cases,Missed  Detection=number  of  GBM  samples  uncorrectly  classifiedtotal  number  of  sample  cases,Accuracy=number  of  GBM  and  normal  samples  correctly  classifiedtotal  number  of  sample  cases.

Moreover, receiver operating characteristic (ROC) curves and the associated area under the curve (AUC) values were computed to assess the discrimination between GBM and normal areas [40]. The results of the performance metrics reflected the succeeding GMM features for discrimination between GBM and normal area. Note that the training data set of the normal brain tissue regions represent different normal regions within the MR image.

## 3. Experimental Results and Discussions

### 3.1. Segmentation of the GBM

**Figure 4:**
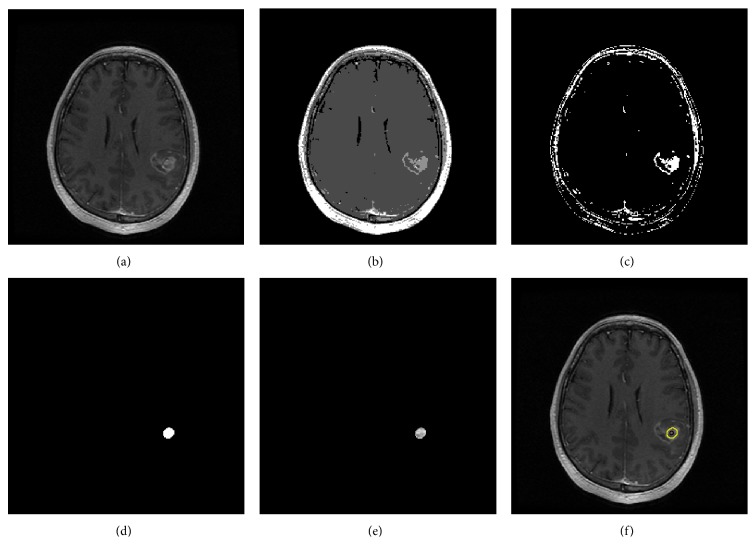
GBM detection by segmentation and morphology operations: (a) T1-MR image, (b) image segmented by four levels, (c) range of GBM gray level conserve, (d) filtering of (c), (e) raw GBM data detected, and (f) GBM located on the brain image.

GBM tumor tissue was detected using the multithresholding segmentation based on Otsu's technique and the morphology operation to obtain only the abnormal brain regions in robust term.  Figure 4 shows GBM tumor segmented using several steps. The process of tumor detection from MR images may appear to be a difficult task as MR images contained some areas which have a similar range of gray color (Figure 4(b)). Morphology operators or filtering was necessary to remove noise like the boundary of skull and brain (Figures 4(c) and 4(d)). Then GBM was detected and located (Figures 4(e) and 4(f)).

### 3.2. GMM Feature Extraction and Classification

**Figure 5:**
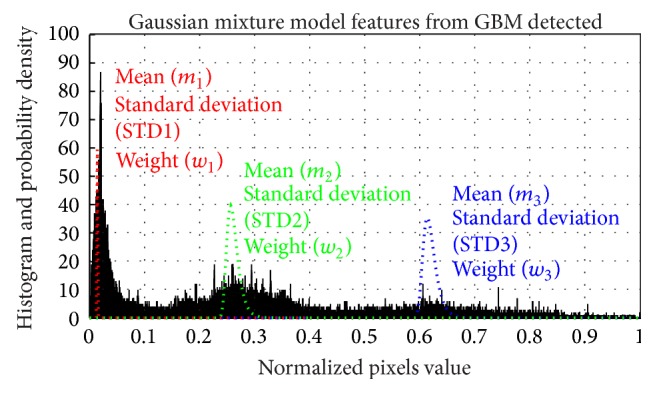
GMM curve fitting: example of GBM based GMM features.

Three GMM curve fittings based on the histogram analysis showed three components of GBM (see Figure 5). Three Gaussian components were chosen based on the empirical metrics which showed three components of GBM. GMM features were shown to be feasible for discriminating between GBM and normal brain.

Table 1 shows a comparative study between the three modes of MR images based on the classifier accuracy, false alarm, and missed detection. These metrics represented the highest performance using NB classifier with the classification accuracy range between 94.00 and 97.00%, false alarm range between 0.00 (which means that the normal area samples were correctly classified without error) and 5.89%, and missed detection range between 0.00 (which means that the GBM samples were correctly classified without error) and 2.95%. This latter value of missed detection represented the one GBM sample from 17 that was incorrectly classified (or classified as normal area).

GMM features were reduced with a PCA, which accounted for 97% of the cumulative variance from these features.  Table 2 shows the performance metrics of the classifier accuracy based on the PCA. Clearly, the accuracy was decreased in the two MRI sequences T1-WI and T2-WI with the best performance achieved using BN classifier, where the accuracy ranged between 73.52 and 79.41%, false alarm ranged between 2.94 and 8.82%, and missed detection is 17.64%. In FLAIR sequence, PNN model showed highest value (94.11%, 0.00%, and 5.88%) of accuracy, false alarm, and missed detection, respectively.

**Figure 6:**
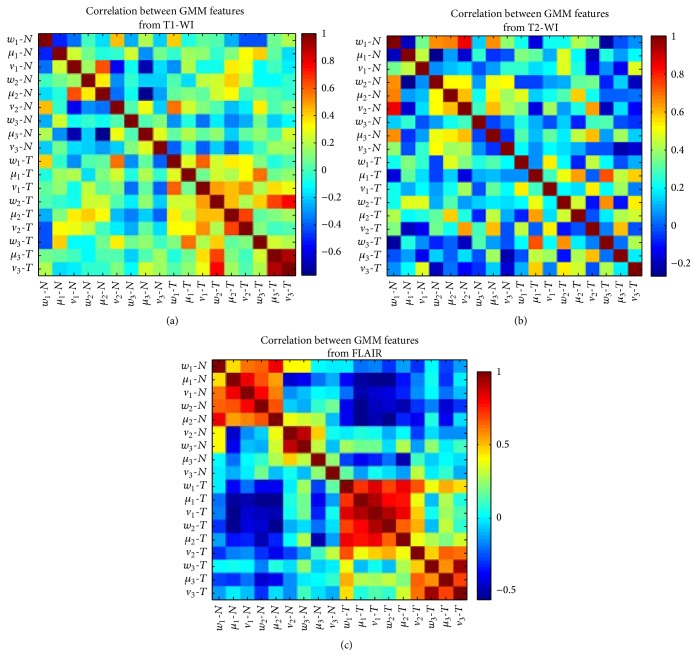
Heat map with correlation coefficients between GMM features: *w*, *μ*, and *v* are the weight, average, and variance, respectively; *N* and *T* are the index of normal and tumor (GBM) areas, respectively.

Clearly, the accuracy decreased in T1-WI and T2-WI which reflected the lack of PCA features. In other words, GBM features provided from GMM were likely independent which was represented by the decrease accuracy value when we applied the PCA, while FLAIR sequence showed a similar value of 94.11% with GMM and PCA features which represent the correlation between the features. This is also represented by the highest correlation value of GMM features in the FLAIR sequence (Figure 6), while the heat map of correlation shows a less value in T1-WI and T2-WI sequences.

Another aspect of the classification was considered by using all data based on 51 images (entire GBM) including 17 images T1-WI, T2-WI, and FLAIR. Table 1 shows the classifier accuracy decrease to 86.27% with 2.94% false alarm and 10.78% missed detection using BN classifier. Clearly, the discrimination between GBM and normal brain tissue using single MR sequence was better than using all together. The classifier accuracy decreased to 68.62% (see Table 2) when we applied the PCA which proved again that the GMM features had no redundancy information in T1-WI and T2-WI and are better to be used for the discrimination of GBM from normal brain based on single MRI sequence. Note that BN classifier showed a better performance than SVM and PNN classifier model.

**Figure 7:**
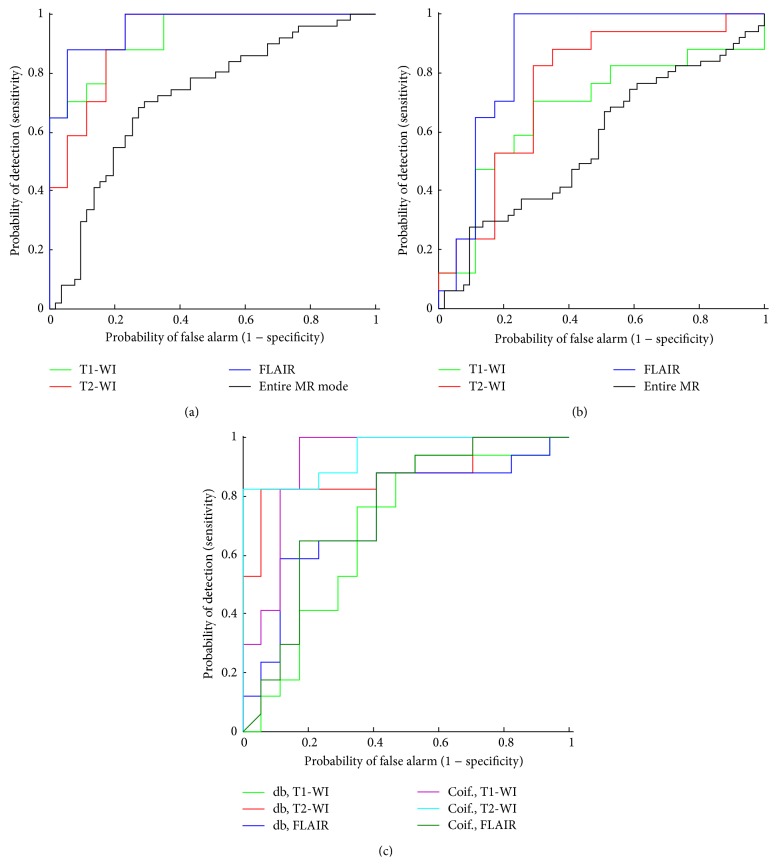
ROC curves of GBM and normal area discrimination based on T1-WI, T2-WI, FLAIR, and entire MR mode (T1-WI, T2-WI, and FLAIR, 51 images): (a) GMM features, (b) PCA features, and (c) Daubechies (*db1*) and Coiflets (*coif1*) wavelet features.

Moreover, ROC curves and the associated AUC have been computed.  Figure 7 shows the ROC curves to evaluate the quality of a classifier.  Table 4 shows that AUC values of 95.85% based on GMM features are better than PCA (AUC = 86.16%) using FLAIR sequence. Clearly, the AUC obtained by using GMM features were better than those from PCA.

### 3.3. Comparatives and Discussions

A comparative study was employed using wavelet based feature [29–31]. Two wavelets, namely,* Daubechies (db1)* and* Coiflets (coif1)*, were considered [41, 42]. Three quantified functions were computed, namely, average, standard deviation, and entropy. Classifier accuracy based on the wavelet features showed the highest accuracy value of 88.23% (*coif1*) in T2-WI using SVM and PNN, 79.41% (*coif1*) in T1-WI, and 70.58% (*db1*) in FLAIR sequence using PNN and NB (see Table 3). ROC curves were associated with AUC based on wavelet feature (*coif1*) with range value 74.50−94.46% better than those based on wavelet feature (*db1*) which showed values of 68.51–87.54% (Figure 7(c) and Table 4). We note that the main goal of the feature extraction using discrete wavelet transform technique is that the approximation coefficients usually contain the most important information (low frequency). Hence they constitute one part of the extracted features and another represented by the critical information from the high-frequency part (details). In comparison, GBM identification is more promising using the GMM features with highest accuracy classifier.

This work is the first focusing on the robust GBM characteristics using the GMM features, while previous literature showed the efficiency of the texture and statistical features analysis to discover brain tumors heterogeneity [24–27, 38]. Through the texture analysis we find that this changes depending on the size and number of the pixels in the determined region. For example, cooccurrence matrix provides valuable information about the relative position of the neighboring pixels in an image. It has been proved that the texture descriptors improve performance of diagnosis where texture is an important source of image characteristics [24, 25]. However, GBM region can be easily distinguished from normal brain areas using the GMM features because we determined the number of Gaussian components (three components in this work [43]), and the number or swap of pixels did not affect the accuracy result. In other words, the number of features is independent of the pixels in the brain tumor image. All GBM tumor regions being diagnosed following MR imaging, the big advantage of this technique derives from the robust processing of MRI data to the final decision.

Obviously, neuroradiologists are becoming more and more important players to early diagnose GBM. Our vision is to integrate engineering based methods as described in daily practice to enhance radiologists' performance beyond their routine “vision.” In particular in utterly devastating diseases, like GBM, improvement in any medical specialty involved is of utmost essence.

As a limitation, this study is based on a single cohort design and subject to its respective limitations. Second, the number of 17 patients limited our analysis of the GBM heterogeneity. However, there is currently no consensus on how to assess GBM heterogeneity. Third, the goal of this study was to analyze the number of images features to determine the efficiency of the GMM features as prognostic indicator. And last, the entire algorithm worked and collected data automatically.

## 4. Conclusions

This paper implements the GMM features extracted from GBM tumor using MR images to assist in radiologic GBM heterogeneity recognition. By a comparative study of other features types as PCA and wavelet, this technique resides in its ability to detect GBM automatically with high accuracy performance which was difficult previously. Turning to the future, it is the authors' intentions to extend this work to implement a full algorithm integrated on MRI equipment in order to identify the GBM robustly using GMM features.

## Figures and Tables

**Table 1:** Performance metrics (%) based on the GMM features.

Classifier	Sequence	Accuracy	False alarm	Missed detection
NB	T1-WI	97.05	2.95	0
	T2-WI	97.05	0	2.95
	FLAIR	94.11	5.89	0
	^*∗*^Entire GBM	86.27	2.94	10.78

SVM	T1-WI	70.58	0	29.41
	T2-WI	64.70	5.88	29.41
	FLAIR	67.64	2.94	29.41
	^*∗*^Entire GBM	66.66	4.90	28.43

PNN	T1-WI	94.11	5.89	0
	T2-WI	70.58	11.76	17.64
	FLAIR	94.11	2.94	2.94
	^*∗*^Entire GBM	86.27	2.94	10.78

^**∗**^Entire GBM refers to T1-WI, T2-WI, and FLAIR features combined together.

**Table 2:** Performance metrics (%) based on the PCA features.

Classifier	Sequence	Accuracy	False alarm	Missed detection
NB	T1-WI	73.52	8.82	17.64
	T2-WI	79.41	2.94	17.64
	FLAIR	82.35	5.88	11.76
	Entire GBM	68.62	15.68	15.68

SVM	T1-WI	55.88	0	44.11
	T2-WI	61.76	8.82	29.41
	FLAIR	85.29	0	14.70
	Entire GBM	35.29	20.58	44.11

PNN	T1-WI	61.76	8.82	29.41
	T2-WI	52.94	14.70	32.35
	FLAIR	94.11	0	5.88
	Entire GBM	51.96	9.80	38.23

**Table 3:** Performance metrics (%) based on the wavelets.

Classifier	Metrics	T1-WI		T2-WI		FLAIR	
		*db1 *	*coif1 *	*db1 *	*coif1 *	*db1 *	*coif1 *
NB	Accuracy	67.64	79.41	82.35	85.29	70.58	64.70
	False alarm	17.64	8.82	8.82	8.88	8.82	14.70
	Missed detection	14.70	8.8235	8.82	8.82	20.58	20.58

SVM	Accuracy	50	76.47	85.29	88.23	58.82	55.88
	False alarm	20.58	14.70	2.94	0	8.82	8.82
	Missed detection	29.41	8.82	11.76	11.76	32.35	35.29

PNN	Accuracy	70.58	79.41	82.35	88.23	70.588	58.82
	False alarm	20.58	20.58	11.76	5.88	20.58	29.41
	Missed detection	8.82	0	5.88	5.88	8.82	11.76

^*∗*^ *db1* and *coif1* are the first order of Daubechies and Coiflet wavelet, respectively.

**Table 4:** Comparison of area under (%) ROC curve between three feature types.

Feature		T1-WI	T2-WI	FLAIR
Wavelets	*coif1 *	91.35	94.46	74.5
	*db1 *	68.51	87.54	73.7

GMM		92.73	91.70	95.85

PCA		67.82	75.43	86.16
