# Corrigendum to “Automated Feature Extraction in Brain Tumor by Magnetic Resonance Imaging Using Gaussian Mixture Models”

**DOI:** 10.1155/2017/3247974

**Published:** 2017-08-13

**Authors:** Ahmad Chaddad, Markus Luedi, Pascal O. Zinn, Rivka Colen

**Affiliations:** ^1^Department of Diagnostic Radiology, University of Texas MD Anderson Cancer Center, 1400 Pressler Street, Houston, TX 77030, USA; ^2^Baylor College of Medicine, 1 Baylor Plaza, Houston, TX 77030, USA

In the article titled “Automated Feature Extraction in Brain Tumor by Magnetic Resonance Imaging Using Gaussian Mixture Models” [1], Markus Luedi, Pascal Zinn, and Rivka Colen were missing from the authors' list. The corrected authors' list is shown above.
